# Supplementary material for: Increased Expression of VANGL1 Is Predictive of Lymph Node Metastasis in Colorectal Cancer: Results from a 20-Gene Expression Signature
Source: J Pers Med. 2021 Feb 14;11(2):126. doi: 10.3390/jpm11020126 (PMC7918343; doi:10.3390/jpm11020126)
Supplement: Supplementary file 1 [file jpm-11-00126-s001.pdf]

Table S1: Logistic Regression Test of the 20 Genes (Analysis – Metastasis)

Gene Name	Obs	Asymptotic Normal (95% Conf. Interval)		Std. Error	Roc Area
		Min	Max		
MTA-1	100	0.40542	0.63545	0.0587	0.5204
LEF-1	100	0.40362	0.64693	0.0622	0.5251
IL2RA	100	0.39427	0.62761	0.0595	0.5109
IL2RB	100	0.45967	0.69520	0.0601	0.5774
EGFR	100	0.41966	0.64898	0.0585	0.5343
MAP2K1	100	0.38429	0.61742	0.0595	0.5009
BUB1	100	0.38234	0.62238	0.0612	0.5024
RHO-A	100	0.44081	0.69991	0.0661	0.5704
CD-44	100	0.40102	0.65346	0.0644	0.5272
CD-133	100	0.41779	0.66115	0.0621	0.5395
PCSK	100	0.46159	0.69542	0.0597	0.5785
SMAD4	100	0.39785	0.62875	0.0589	0.5133
VANGL-1	100	0.52410	0.75561	0.0591	0.6399
FOLH1	100	0.39754	0.63420	0.0604	0.5159
HES-1	100	0.43953	0.67716	0.0606	0.5583
ANXA3	100	0.45440	0.69575	0.0616	0.5751
NOTCH-1	100	0.37348	0.60721	0.0596	0.4903
SMAD2	100	0.46244	0.69243	0.0587	0.5774
PITX2	100	0.40716	0.65075	0.0621	0.5290
TGF-β	100	0.48512	0.68214	0.0501	0.5587

Table S2: Logistic Regression Test of the 20 Genes (Analysis – Stage)

Gene Name	Obs	Asymptotic Normal (95% Conf. Interval)		Std. Error	Roc Area
		Min	Max		
MTA-1	100	0.40542	0.63545	0.0587	0.5204
LEF-1	100	0.40094	0.63352	0.0593	0.5172

IL2RA	100	0.43423	0.66192	0.0581	0.5481
IL2RB	100	0.41213	0.64155	0.0585	0.5268
EGFR	100	0.39573	0.62750	0.0591	0.5116
MAP2K1	100	0.33020	0.55762	0.0580	0.4439
BUB1	100	0.38855	0.61826	0.0612	0.5034
RHO-A	100	0.45397	0.68986	0.0602	0.5719
CD-44	100	0.36723	0.59992	0.0594	0.4836
CD-133	100	0.41237	0.64292	0.0588	0.5276
PCSK-7	100	0.29640	0.52491	0.0583	0.4107
SMAD4	100	0.40131	0.63034	0.0584	0.5158
VANGL-1	100	0.49699	0.72136	0.0572	0.6092
FOLH1	100	0.41903	0.64908	0.0587	0.5341
HES-1	100	0.42989	0.65986	0.0587	0.5449
ANXA3	100	0.40782	0.64106	0.0595	0.5244
NOTCH-1	100	0.41026	0.64022	0.0587	0.5252
SMAD2	100	0.44960	0.67740	0.0581	0.5635
PITX2	100	0.38402	0.61438	0.0588	0.4992
TGF-β	100	0.40612	0.63675	0.0588	0.5214
